# Multimorbidity and Mental Health: The Role of Gender among Disease-Causing Poverty, Rural, Aged Households in China

**DOI:** 10.3390/ijerph17238855

**Published:** 2020-11-28

**Authors:** Chen Jiao, Anli Leng, Stephen Nicholas, Elizabeth Maitland, Jian Wang, Qinfeng Zhao, Lizheng Xu, Chaofan Gong

**Affiliations:** 1 Centre for Health Management and Policy Research, School of Public Health, Cheeloo College of Medicine, Shandong University, Jinan 250012, China; jiaochen987@163.com (C.J.); zhaoqinfeng6@163.com (Q.Z.); 2 NHC Key Lab of Health Economics and Policy Research, Shandong University, Jinan 250012, China; 3 School of Political Science and Public Administration, Institute of Governance, Shandong University, 72 Binhai Rd, Qingdao 266237, Shandong, China; lenganli@sdu.edu.cn; 4 Australian National Institute of Management and Commerce, 1 Central Avenue, Australian Technology Park, Sydney, NSW 2015, Australia; stephen.nicholas@newcastle.edu.au; 5 Guangdong Institute for International Strategies, Guangdong University of Foreign Studies, 2 Baiyun North Avenue, Guangzhou 510420, Guangdong, China; 6 School of Economics and School of Management, Tianjin Normal University, No. 339 Binshui West Avenue, Tianjin 300387, China; 7 Newcastle Business School, University of Newcastle, Newcastle, NSW 2308, Australia; 8 School of Management, University of Liverpool, Chatham Building, Chatham Street, Liverpool L697ZH, UK; e.maitland@liverpool.ac.uk; 9 Dong Fureng Institute of Economics and Social Development, Wuhan University, No. 54 Dongsi Lishi Hutong, Dongcheng District, Beijing 100010, China; wangjian993@whu.edu.cn; 10 Center for Health Economics and Management, Economics and Management School, Wuhan University, Luojia Hill, Wuhan 430072, China; 11 The George Institute for Global Health, Sydney, NSW 2052, Australia; lizheng.xu@unsw.edu.au; 12 UNSW Medicine, UNSW Sydney, Sydney, NSW 2052, Australia; 13 Center for Digital Health, School of Medicine, Stanford University, Palo Alto, CA 94305, USA

**Keywords:** multimorbidity, mental health, older-age adults, gender, disease-causing poverty

## Abstract

(1) Background: The association between multimorbidity and mental health is well established. However, the role of gender in different populations remains unclear. Currently, China is facing an increased prevalence of multimorbidity, especially in its disease-causing poverty population. The present study explores the gender-based differences in the relationship between multimorbidity and mental health using data from the rural, disease-causing poverty, older-age population in Shandong province, China, as a case study. (2) Methods: The data were obtained from the survey on the health and welfare of disease-causing poverty households in rural Shandong province. We identified 936 rural participants who were over 60 years old from disease-causing poverty households. The mental health status was measured using the Kessler Psychological Distress Scale (K10) instrument. Using a multivariable linear regression model, including the interaction of gender and multimorbidity, gender differences in the association between multimorbidity and mental health were explored. (3) Results: Multimorbidity was a serious health problem in rural, disease-causing poverty, older-age households, with the prevalence of multimorbidity estimated as 40% for women and 35.4% for men. There was a strong association between multimorbidity and mental health, which was moderated by gender. Women had higher K10 scores than men, and the mean K10 score was highest in women with three or more chronic diseases. Compared with men, women with multimorbidity had a higher risk of mental health problems. (4) Conclusions: The prevalence of multimorbidity in older-age rural disease-causing poverty subpopulations is a severe public health problem in China. The association between multimorbidity and mental health differed by gender, where multimorbid women suffered an increased mental health risk compared with men. Gender differences should be addressed when delivering effective physical and mental healthcare support to disease-causing poverty, older-age, rural households.

## 1. Introduction

Global aging is a defining social trend in the 21st century. In 2020, the World Health Organization’s (WHO) global health and research priority is healthy aging [1]. At present, the main public health and individual medical problem faced by older-age adults is chronic diseases. The prevalence of two or more coexisting multiple chronic diseases [2], or multimorbidity, has become a major challenge for chronic disease control [3] and a common phenomenon in people aged over 60 years old [4]. Multimorbidity impairs an individual’s overall health and imposes a heavy financial burden on families, the health system, and the society [5]. In 2013, it was reported that 28% of Americans suffered from multimorbidity, and multimorbidities accounted for 66% of total healthcare spending [6]. According to a Canadian study, the incidence of two or more chronic diseases in the older-age adults was as high as 76.5% [7], and a Canadian community survey found the prevalence of multimorbidity significantly increased between 2008 and 2014 [8]. The Australian National Health Survey reported almost 80% of Australians aged 65 years or older had three or more chronic conditions [9]. China is also facing severe health challenges because of the rapid growth in the size of the older population. A Chinese study found that more than half those aged 70 or older were multimorbid [10] and 80% of those over 75 years old had three or more chronic diseases [11]. Multimorbidity is not only a function of an aging population, but related to population health factors, such as a high body mass index, quality of life, and the population’s socioeconomic status [12]. The worldwide prevalence of chronic diseases in older-age adults poses important public health problems, including mental health disorders, globally and in China [13].

Mental health disorders are a serious problem in their own right and common in people with multimorbidity. About 20% of older people have reportedly experienced some type of mental health problem. While we pay attention to physical functioning and disease conditions, we also need to be aware of mental health illnesses, especially depression, which is expected to become the leading cause of disability in developed countries in 2030 and the second leading cause of disability in the world. The epidemiological literature on multimorbidity has consistently found high rates of affective and anxiety disorders in those with multiple diseases [14]. Multimorbid people are twice as likely to be psychological distressed as people without multimorbidity [15]. Comorbidity between chronic diseases and affective disorders has been reported in numerous studies across diverse countries, including Canada [16], the Netherlands [17], Germany [18], the UK [19], and Australia [20]. In the US, the National Comorbidity Survey (NCS) [21] and the National Epidemiological Survey on Alcohol and Related Conditions (NESARC) [22] have identified a relationship between multimorbidity and psychological distress. The presence of multimorbidity complicates the question of how chronic diseases are related to outcome variables, such as quality of life or mental health conditions [23]. Studies have revealed that pre-existing chronic diseases significantly contribute to the development of mental health disorders and that pre-existing mental health disorders can also significantly contribute to the development of chronic disease over time. It is very likely that the relationship between physical diseases and mental health is temporally bidirectional [24].

The relationship of multimorbidity and mental health is influenced by two primary factors: gender and the attributes of the subpopulation under study [13]. The results of gender differences in the relationship between multimorbidity and mental health have been inconsistent. A survey of the older adults in Taiwanese urban communities revealed no significant gender differences between chronic diseases and mental health [25], while a study of older adults in Suzhou indicated that men with chronic diseases had more mental disorders than women [26]. The explanation of lower mental disorders in multimorbid women may partly depend on females more widely participating in social activities, which makes them feel happy and relaxed. Women are also better at communicating and may better channel bad emotions than men. A study of the older adults in rural China revealed a significant association between multimorbidity and lower mental health problems for women, but not for men [27]. One explanation is that older-age female patients are considered a disadvantaged group in society, often suffering negative emotions and loneliness; this can lead to women with chronic diseases having more mental disorders than men.

Several studies identified the most important risk factors for rural, aged patients’ mental health were self-assessed inadequate or arrears financial status [28]. International research showed that the increase in debt or debt level was related to the subsequent mental disorders [29], confirming that individuals’ physical well-being and financial condition are closely related to mental health. Chronic diseases impose financial pressure on families, pushing many households, especially those with multimorbid aged members, into poverty. Disease leads to poverty by two major pathways: the death or disability of a household income earner due to disease, and, second, high medical-related costs related to disease treatment. Disease-causing poverty households face out-of-pocket (OOP) medical expenses in excess of 40% of household income after expenditure on food, which reduces non-health household expenditure below the level required for necessities [30]. Disease-causing poverty is a serious problem in rural China, with an estimated 30 million living in poverty, including 42% in disease-causing poverty [31,32]. At-risk disease-causing poverty households often have low educational attainment, are dependent on agricultural income, experience a high dependency ratio, contain members in poor health, and face existing debts or no savings. We focus on the mental health of this vulnerable, but neglected, rural, multimorbid, disease-causing poverty, older-age subpopulation.

There is little information on gender differences in the mental health status of Chinese disease-causing poverty, rural, older adults suffering chronic diseases. Although chronic diseases in older adults are common [33], the relationship of multimorbidity and mental health disorders between different genders over time is not well understood. This study investigated the association in multimorbidity and status of mental health between different genders in disease-causing poverty, older-age households. An understanding of the relationship between multimorbid, rural, older-age adults in disease-causing poverty households and their mental health will help healthcare managers design more appropriate and targeted interventions. To address this gap in the literature, first, we evaluated the association between multimorbidity and mental health among the rural, disease-causing poverty, older-age adults in Shandong Province, China. Second, we explored whether gender impacted the association between multimorbidity and mental health among this rural, disease-causing poverty subpopulation.

## 2. Materials and Methods

### 2.1. Data and Sample

We took a cross-sectional, multistage, unweighted, stratified, random sample based on the Shandong Provincial Health Poverty Alleviation Information System, which contained data on all the disease-related poverty households in Shandong province. The inclusion criteria were households with a per capita income during the most recent twelve months less than RMB683 (€87 or U.S. $104), who suffered from one or more serious disease with high medical costs. In step one, our survey was carried out in 3 cities, Taian, Binzhou and Zaozhuang, in Shandong province; in step two, 10 townships in the 3 cities were selected; in step three, 43 rural village committees were selected; in step four, 30 households in each rural village committee were selected. A total of 1264 households were included in the investigation. One eligible household member over 60 years old, without cognitive impairments and with normal daily living activities and social interactions, normal problem-solving ability and normal memory, was selected to be the survey respondent. Face-to-face interviews were made using electronic questionnaires on tablet personal computers. After deleting missing values, among all the 1264 interviewees, a total of 936 older-age participants completed the questionnaire, with a mean age of 73.1 ± 6.73 years. All respondents were informed about the survey aims, assured anonymity, and provided informed consent. The project was approved by the Public Health Ethics Committee at Shandong University School of Public Health (Grant No. 20190406). Written informed consent was obtained from all participants prior to research.

### 2.2. Key Variables, Multimorbidity

The key variable was multimorbidity, which was measured as the number of self-reported chronic diseases, selected from hypertension, diabetes, coronary heart disease, chronic bronchitis, cancer, asthma, chronic pharyngitis, infarction cardiac, stroke, gastric ulcer, and other chronic diseases. The variable of multimorbidity was divided into four categories (0, 1, 2, and ≥3 diseases).

### 2.3. Mental Health

The Chinese version of the nonspecific psychological Kessler Psychological Distress Scale (K10) was used to assess respondents’ mental health [34,35]. The scale contains 10 items (tired, nervous, severely nervous, helpless, restless, severely restless, depressed, everything is difficult, hopeless, and worthless), each divided into 5 levels. Each item was scored as 1–5 in according with its frequency in the past four weeks, with 1 meaning “none of the time”, 2 meaning “little of the time”, 3 meaning “some of the time”, 4 meaning “most of the time”, and 5 meaning “all of the time” [35]. With total scores ranging from 10 (indicating no psychological distress) to 50 (indicating severe psychological distress), K10 focuses on anxiety and depression, which are commonly used indicators to measure psychological problems [36]. Following the literature, scores were divided into four levels: 10–15 (good mental health group), 16–21 (general mental health group), 22–29 (poor mental health group), and 30–50 (severe mental disorder group) [37].

### 2.4. Sociodemographic Variables

Demographic characteristics were collected comprising gender (female, male), age (60–69 years old, 70–79 years old, ≥80 years old), education attainment (illiteracy, primary school, middle school, or above), marital status (yes: married or living together, no: unmarried, separated, divorced, or widowed), work status (yes: family farming, family breeding, employed by others or units, part-time jobs without fixed employers, self-employed households, freelance jobs without employers or others, no: no work), medical insurance (yes: had medical insurance, no: no medical insurance).

### 2.5. Statistical Analysis

We performed descriptive statistical analysis on all variables. The results were described using either means and standard deviations or numbers (proportion). Gender differences in the sociodemographic characteristics were calculated using the Student’s *t*-test (for continuous variables) or chi-square test (for categorical variables). ANOVA was used to compare differences in multimorbidity and K10 scores. Multivariable linear regression models, with K10 scores as the dependent variable, were estimated for women and men separately to assess the association between multimorbidity status and mental health after controlling for influential potential factors. Odds ratio (OR) expressed the strength of the association between exposure factors and diseases. Gender differences in the association between multimorbidity status and mental health were examined by adding gender to the multimorbidity interaction term (gender* multimorbidity status) in the model. All data were analyzed using STATA 14.0 (Stata Corp, College Station, TX, USA), with results considered statistically significant when *p*-values were less than 0.05.

## 3. Results

### 3.1. Respondents’ Characteristics

Descriptive statistics for the sample are shown in Table 1. From the total of 936 older-age adults, women accounted for 52.9% and men for 47.1% of the sample; men comprised a higher proportion of the population who were middle-aged older adults (*p* < 0.05), more highly educated (*p* < 0.001), and married (*p* < 0.001) than women. The proportion of women who worked (*p* < 0.001) and had medical insurance (*p* < 0.05) was significantly lower than that of men. The proportion of women with multimorbidities was 40% (*p* < 0.001), which was slightly higher than that of men. The prevalence of not having a disease was only 4.6% for women and 5.7% for men, and the prevalence of having one disease was 55.4% for women and 58.9% for men. The mean (SD) K10 score for women was 28.13 ± 2.35, which was significantly higher than for men (*p* < 0.001).

### 3.2. Comparison of Mental Health between Categories of Multimorbidity Status

**Figure 1:**
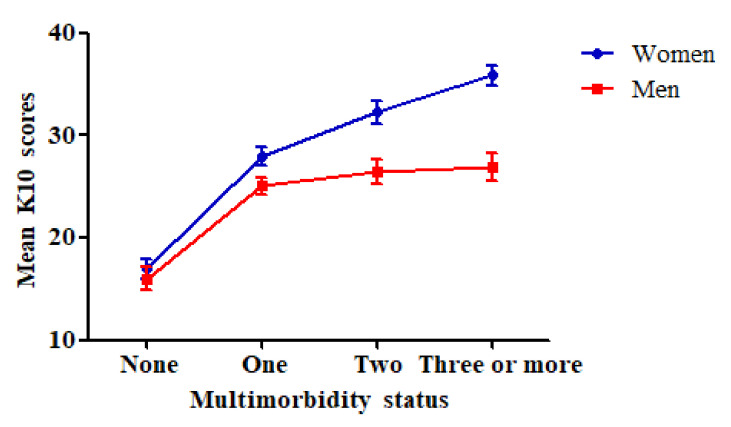
Mean K10 score for each multimorbidity status by gender.

Table 2 reports the results of comparing K10 scores between the different categories of multimorbidity status in women and men separately. There were significant gender differences in mean K10 scores between the four multimorbidity conditions. For women, post hoc test results showed that the without a disease group had better mental health (*p* < 0.001), while the having two or more diseases group (*p* < 0.001) had worse psychological distress than the one disease group. Men without a disease group had better mental health (*p* < 0.001) than men having one disease group, but there was no significant difference between the men having two or more diseases group and the men having one disease group. Multimorbid males were in the poor mental health group, but females with multimorbidity were in the severe mental disorder group. As shown in Figure 1, the mean K10 score was highest in women with three or more diseases, and the score was lowest in men without a disease.

### 3.3. Association between Multimorbidity and Mental Health and Its Gender Differences

**Figure 2:**
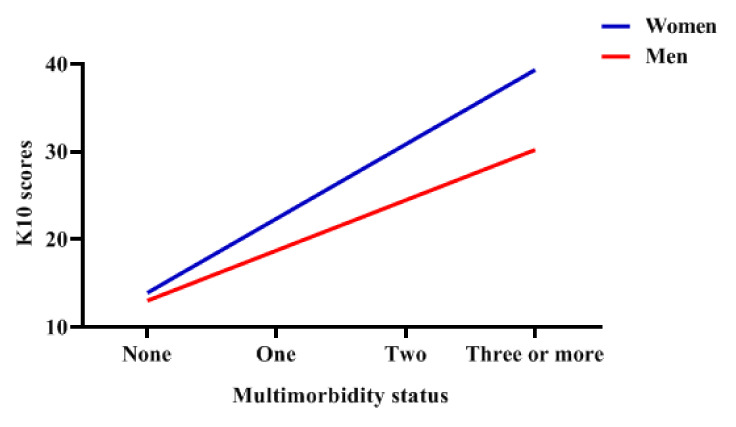
The role of gender among the association between multimorbidity status and mental health.

Table 3 summarizes the multivariable linear regression models of mental health measured by K10. First, Table 3 reports the separate regression models for women (Model I) and men (Model II). Compared with the group having one disease, the group having no disease reported significantly better mental health in both women (OR 0.506, 95% CI 0.361–0.667) and men (OR 0.144, 95% CI 0.092–0.223). However, for the multimorbidity group, multimorbidity had a significant negative effect on mental health in women (Model I), but not in men (Model II). More importantly, the difference between women and men reported in interaction Model III was significant. As shown in Figure 2, the result of simple slope test showed that there was a strong association between multimorbidity and mental health, which was moderated by gender. Women with multimorbidity had a higher risk of mental health problems. In addition, the results showed that age, education attainment, marital status, work status, and the availability of medical insurance had a significant impact on respondents’ mental health (*p* < 0.05). The young-aged (60–69 years old) adults had more serious mental disorders. The older adults with a high level of education, married, and having a job and medical insurance had significantly better mental health.

## 4. Discussion

For the vulnerable rural, disease-causing poverty, old-age subpopulation, we investigated the gender differences in the association between multimorbidity and mental health. The highest mean K10 score was achieved by women with multimorbidity status, and the lowest mean K10 score was achieved by men having no diseases. After controlling potential confounders, gender had a significant effect on the association between multimorbidity and mental health.

It is well known that chronic diseases negatively impact the well-being and mental health of older adults [38]. Results from both cross-sectional and longitudinal studies have confirmed that older people with multimorbidity reported more symptoms of mental health disorder, as compared with those without chronic diseases [39]. The empirical evidence generally supports the hypothesis that multimorbidity increases the risk of experiencing mental health disorders and that the more serious the chronic diseases, the more serious the mental health disorders. We performed multivariable linear regression analysis to estimate the effects of the interactions of gender by multimorbidity on mental health for a neglected rural, disease-causing poverty, older-age subpopulation. We found women suffered from the negative effects of multimorbidity on mental health even after adjusting for other potential influential factors. One possible explanation for gender differences in multimorbidity was that women suffering disease-causing poverty might be more sensitive to disease than men. Further, economic distress could increase the risk of women suffering from psychological distress from multimorbidities.

In China, the vicious cycle of disease and poverty remains a challenge. Disease has short-term and long-term effects on poverty, where poverty also affects the utilization of medical services, which in turn increases health vulnerability. Taking gender into consideration is essential for understanding health conditions. The studies of gender differences in the health characteristics of those suffering diseases have been investigated in many countries [40]. For example, a previous study using rural data found that women with diabetes had a higher prevalence of affective and anxiety disorders than corresponding men [41]. Many studies have found that older women are usually more affected by poor mental health than older men [42]. Moreover, gender differences were found in terms of the prevalence of anxiety disorders in epidemiologic surveys in Chinese older people [43].

Our study found that the rural women with low levels of education, single marital status, and not having medical insurance were more likely to have a poor mental health status than men. Health outcomes are influenced by social and economic factors that, in turn, are influenced by cultural and political conditions in society [33]. In rural China, women have a lower social status and older women have a lower socio-economic status than men, which may delay identification and management of mental health issues and increase the prevalence and severity of psychological distress in women [44]. Lower socio-economic status and education level mean women experience more stressful life events and fewer psychological and social coping resources. Many women cannot find a good way to actively cope with mental stress, impacting their self-efficacy and self-identification, especially in disease-causing poverty households. Studies have shown that women’s mental health is more affected by marriage and family than men. The destruction of a marriage will inevitably have a significant impact on a woman’s physical and mental health [45], with single older women more likely to be lonely, restless, and helpless. Our study found that the existence of medical insurance had a significant impact on the mental health of women. One conclusion is that the poverty households unable to afford insurance did not receive adequate treatment due to out-of-pocket medical expenses, and physical discomfort due to multimorbidities further induced bad emotions. Our research is consistent with evidence indicating that differences in neurochemical and brain structure between different genders may be associated with different gender-specific psychiatric problems [46]. Older-age women’s mental problems in disease-causing poverty households were triggered by socio-economic conditions, marital status, and health insurance. Such scenarios obviously call into question access to health and social care systems.

As multimorbidity and mental health problems become increasingly common in China, there is an increased challenge to provide new ways to deliver healthcare [47]. Gender analysis is a key way to understand the healthcare of multimorbid women, especially older-age women in disease-causing poverty households, and to tailor interventions to prevent, monitor, and treat physical and mental illness [48]. Generally speaking, the mental health of women is especially complex, affected by multiple factors, including emotional care, social respect, and economic and financial assistance [10]. Women, especially single, older-age women, are disadvantaged relative to men in Chinese society, where women’s health, psychological and social needs are frequently neither recognized nor met [49]. We recommend strengthening the construction of the social support system for disease-causing poverty households, especially households with older-age women. Second, multimorbidities and mental health issues are not just a medical problem but a reflection of a household’s socio-economic circumstances. The government should establish poverty reduction programs, protecting families from poverty and the mental stress of poverty. Third, given the differential quality in urban and rural health facilities [45], the government should improve rural health services, especially for those with multimorbidities. Fourth, old-age care facilities are not well developed in China, creating a gap in mixed nursing and long-term care services. Government should provide mixed nursing and old-age care for disease-causing poverty households, especially with single female members. Fifth, to reduce disease-creating poverty, reforms to China’s national health insurance scheme should provide a safety net for poor households [50]. Sixth, children and relatives have been the most important source of support and care for the older adults, especially sustaining the mental health of the older adults. The one-child policy, smaller families, and the migration of children to urban areas for work have weakened family care for the physically and mentally ill older adults. This gap will need to be filled by government services, especially for multimorbid poor households with physical and mental health problems.

There are several limitations in our study. First, this was a cross-sectional study, the relationship between multimorbidity and mental health cannot be interpreted as cause and effect. Longitudinal studies are needed in the future to explore this causal relationship. Second, our sample consisted of disease-causing poverty, older-age households living in Shandong, an eastern China province. While Shandong may be representative of east coast, industrial, high-income provinces, studies of western and southern, less-developed, more agricultural provinces are required.

## 5. Conclusions

In this population-based study, we found that the multimorbid older-age adults in disease-causing poverty households were more likely to have poor mental health. Gender mattered. Rural, older-age women with low levels of education, single marital status, and not having medical insurance were more likely to have a poor mental health status than men. We recommend the development of effective gender-specific multimorbidity healthcare programs, providing physical and mental health support.

## Figures and Tables

**Table 1:** Sociodemographic and health-related conditions by gender (*n* = 936).

Variable	Total (*n* = 936)	Women (*n* = 495)	Men (*n* = 441)	*p*-Value
	*n* (%)	*n* (%)	*n* (%)	
Age				0.034 ^b^
60–69	214 (22.9)	121 (24.4)	93 (21.1)	
70–79	619 (66.1)	318 (64.2)	301 (68.2)	
≥80	103 (11.0)	56 (11.4)	47 (10.7)	
Education attainment				<0.001 ^b^
None	334 (35.7)	221 (44.6)	116 (26.3)	
Primary school	336 (35.9)	158 (31.9)	181 (41.0)	
Middle school or above	266 (28.4)	116 (23.5)	144 (32.7)	
Marital status				<0.001 ^b^
Yes	504 (53.8)	257 (51.9)	247 (56)	
No	432 (46.2)	238 (48.1)	194 (44)	
Work status				<0.001 ^b^
Yes	263 (28.1)	21 (4.2)	242 (54.9)	
No	673 (71.9)	474 (95.8)	199 (45.1)	
Medical insurance				0.042 ^b^
Yes	630 (67.3)	292 (66.2)	338 (68.3)	
No	306 (32.7)	149 (33.8)	157 (31.7)	
Multimorbidity				<0.001 ^b^
None	48 (5.1)	23 (4.6)	25 (5.7)	
1	534 (57.1)	274 (55.4)	260 (58.9)	
2	287 (30.7)	151 (30.5)	136 (30.8)	
≥3	67 (7.1)	47 (9.5)	20 (4.6)	
K10, mean ± SD	25.68 ± 3.24	28.13 ± 2.35	23.72 ± 2.96	<0.001 ^a^

SD, standard deviation; K10, 10-item Kessler Psychological Distress Scale; ^a^ *t*-test, ^b^ Chi-square test.

**Table 2:** Comparisons of K10 scores between categories of multimorbidity status (*n* = 936).

Variable	None	One Disease	Two Diseases	Three or More Diseases	*p*-Value ^a^
Women					
K10, mean ± SD	16.92 ± 2.16	27.93 ± 1.98	32.28 ± 2.56	35.85 ± 2.21	<0.05
Men					
K10, mean ± SD	15.98 ± 2.44	25.05 ± 1.86	26.44 ± 2.82	26.96 ± 2.99	<0.05

SD, standard deviation; ^a^ one-way ANOVA test for differences of multimorbidity status.

**Table 3:** Multivariable linear regression models for association between multimorbidity status and mental health and its gender difference.

Variable	Model I Women		Model II Men		Model III	
					Interaction: Gender × Multimorbidity	
	OR	95% CI	OR	95% CI	OR	95% CI
Age (Reference: ≥80)						
60–69	1.853 *	(1.009, 3.403)	1.379 *	(1.059, 1.795)	1.541 *	(1.082, 2.191)
70–79	1.790 *	(1.299, 2.466)	1.167	(0.734, 1.792)	1.513 *	(1.092, 1.931)
Education (Reference: None)						
Primary school	0.772	(0.581, 1.027)	0.629	(0.270, 1.465)	0.636	(0.401, 1.009)
Middle school or above	0.687 *	(0.490, 0.962)	0.419 *	(0.295, 0.596)	0.577 *	(0.403, 0.828)
Marital status (Reference: Single)						
Married	0.562 *	(0.384, 0.823)	0.421 *	(0.287, 0.598)	0.504 *	(0.385, 0.660)
Work (Reference: None)						
Yes	0.445	(0.292, 1.067)	0.293 *	(0.185, 0.463)	0.304 *	(0.189, 0.489)
Medical insurance (Reference: None)						
Yes	0.680 *	(0.478, 0.965)	0.504 *	(0.385, 0.660)	0.566 *	(0.381, 0.841)
Multimorbidity (Reference: 1)						
None	0.506 *	(0.361, 0.667)	0.144 *	(0.092, 0.223)	0.307 *	(0.172, 0.493)
2	1.372 *	(1.049, 1.682)	1.009	(0.756, 1.787)	1.242 *	(1.063, 1.782)
≥3	1.517 *	(1.196, 1.937)	1.063	(0.986, 1.242)	1.397 *	(1.165, 1.667)
Women (Reference: Men)					1.342 *	(1.034, 1.761)
Gender × Multimorbidity						
Women × None					0.771	(0.356, 1.672)
Women × Two diseases					1.463 *	(1.176, 1.832)
Women × Three or more diseases					1.681 *	(1.402, 2.011)
Respondents	495		441		936	

OR, odds ratio; CI, confidence interval, * *p* < 0.05; Gender × Multimorbidity = interaction effect between gender and multimorbidity status.

## References

[1] Birk J.L., Kronish I.M., Moise N., Falzon L., Yoon S., Davidson K.W. (2019). Depression and Multimorbidity: Considering Temporal Characteristics of the Associations Between Depression and Multiple Chronic Diseases. Health Psychol..

[2] The World Health Report 2008: Primary Health Care Now More than Ever. https://www.who.int/whr/2008/en/.

[3] Hu X., Huang J., Lv Y., Li G., Peng X. (2015). Status of prevalence study on multimorbidity of chronic disease in China: Systematic review. Geriatr. Gerontol. Int..

[4] Lancet T. (2018). Making more of multimorbidity: An emerging priority. Lancet.

[5] Cui J., Mao F., Wang Z.H. (2016). Comorbidity of common chronic diseases among the elderly in China. Chin. Public Health.

[6] Nilsen W.J., Olster D.H. (2013). News from NIH: Effective behavioral treatments for patients with multiple chronic conditions. Transl. Behav. Med..

[7] Fortin M., Lapointe L., Hudon C., Vanasse A. (2005). Multimorbidity is common to family practice: Is it commonly researched?. Can. Fam. Physician.

[8] Romain A.J., Marleau J., Baillot A. (2019). Association between physical multimorbidity, body mass index and mental health/disorders in a representative sample of people with obesity. J. Epidemiol. Community Health.

[9] Sandison B. (2017). Australian Institute of Health and Welfareÿ. Impact.

[10] Yu Z.-H. (2019). Comparative Study on Mental Health Status of Elderly Hypertensive Patients in Urban and Rural Areas in Shandong Province. Master’s Thesis.

[11] Wang H.X., Wang J.J., Wong Y.S., Wong C.S., Li F.J., Wang P.X., Zhou Z.H., Zhu C.Y., Griffiths S.M., Mercer S.W. (2014). Epidemiology of multimorbidity in China and implications for the healthcare system: Cross-sectional survey among 162,464 community household residents in southern China. BMC Med..

[12] Ahmadi B., Alimohammadian M., Yaseri M., Majidi A., Boreiri M., Islami F., Poustchi H., Derakhshan M.H., Feizesani A., Pourshams A. (2016). Multimorbidity: Epidemiology and Risk Factors in the Golestan Cohort Study, Iran: A Cross-Sectional Analysis. Medicine.

[13] Wells J.C.K., Nesse R.M., Sear R., Johnstone R.A., Stearns S.C. (2017). Evolutionary public health: Introducing the concept. Lancet.

[14] Scott K.M., Browne M.A.O., Mcgee M.A., Elisabeth Wells J. (2009). Mental-physical comorbidity in Te Rau Hinengaro: The New Zealand mental health survey. Aust. N. Z. J. Psychiatry.

[15] Moise N., Khodneva Y., Jannat-Khah D.P., Richman J., Davidson K.W., Kronish I.M., Shaffer J., Safford M.M. (2018). Observational study of the differential impact of time-varying depressive symptoms on all-cause and cause-specific mortality by health status in community-dwelling adults: The REGARDS study. BMJ Open.

[16] Gratzer D., Levitan R.D., Sheldon T., Toneatto T., Rector N.A., Goering P. (2004). Lifetime rates of alcoholism in adults with anxiety, depression, or co-morbid depression/anxiety: A community survey of Ontario. J. Affect. Disord..

[17] De Graaf R., Bijl R.V., Smit F., Vollebergh W.A.M., Spijker J. (2002). Risk factors for 12-month comorbidity of mood, anxiety, and substance use dirorders: Findings from the Netherlands Mental Health Survey and Incidence Study. Am. J. Psychiatry.

[18] John U., Meyer C., Rumpf H., Hapke U. (2004). Smoking, nicotine dependence and psychiatric comorbidity—A population-based study including smoking cessation after three years. Drug Alcohol Depend..

[19] Wells J.E., Browne M.A.O., Scott K.M. (2006). Te Rau Hinengaro: The New Zealand mental health survey: Overview of methods and findings. Aust. N. Z. J. Psychiatry.

[20] Teesson M., Hall W., Lynskey M., Degenhardt L. (2015). Alcohol- and drug-use disorders in Australia: Implications of the national survey of mental health and wellbeing. Aust. N. Z. J. Psychiatry.

[21] Kessler R.C., Chiyu W.T., Demler O., Walters E.E. (2005). Prevalence, severity, and comorbidity of 12-month DSM-IV disorders in the National Comorbidity Survey Replication. Arch. Gen. Psychiatry.

[22] Hasin D.S., Stinson F.S., Ogburn E., Grant B.F. (2007). Prevalence, Correlates, Disability, and Comorbidity of DSM-IV Alcohol Abuse and Dependence in the United States: Results from the National Epidemiologic Survey on Alcohol and Related Conditions. Arch. Gen. Psychiatry.

[23] Rijken M., Kerkhof M.V., Dekker J., Schellevis F.O.G. (2005). Comorbidity of chronic diseases. Qual. Life Res..

[24] Chen C.-M., Lee I.-C., Su Y.Y., Mullan J., Chiu H.-C. (2017). The longitudinal relationship between mental health disorders and chronic disease for older adults: A population-based study. Int. J. Geriatr. Psychiatry.

[25] Li L., Wei L., Shan-Ju H. (2018). Research on mental health status of different types of elderly in urban communities based on correspondence analysis. China Health Stat..

[26] Wu Y. (2012). A Study on the Relationship between Socioeconomic Status and the Equity of the Elderly Health Status: An Evidence-Based Study in Suzhou. Master’s Thesis.

[27] Sheng L., Li L., Jin Z. (2017). Meta-analysis of the influencing factors of mental health of the Chinese elderly. Chin. J. Gerontol..

[28] Niu T.-H., Meng Q.-Y., Song T. (2009). Cumulative logistic regression analysis of factors affecting the mental health of the elderly in rural areas. Chin. J. Health Psychol..

[29] Fitch C., Hamilton S., Bassett P., Davey R. (2011). The relationship between personal debt and mental health: A systematic review. Ment. Health Rev..

[30] Xu K., Evans D.B., Kawabata K., Zeramdini R., Klavus J., Murray C.J.L. (2003). Household catastrophic health expenditure: A multicountry analysis. Lancet.

[31] Wang H.-P., Wang Z.-T., Ma P.-C. (2016). Analysis and Thinking of Poverty Caused by Disease in Rural Areas—Based on Survey Data of 1214 Poor Households Caused by Disease in 9 Provinces and Cities in Western China. Economist.

[32] Leng A., Jing J., Nicholas S., Wang J. (2019). Catastrophic health expenditure of cancer patients at the end-of-life: A retrospective observational study in China. BMC Palliat. Care.

[33] Wang S., Ungvari G.S., Forester B.P., Chiu H.F.K., Wu Y., Kou C., Fu Y., Qi Y., Liu Y., Tao Y. (2017). Gender differences in general mental health, smoking, drinking and chronic diseases in older adults in Jilin province, China. Psychiatry Res..

[34] Sunderland M., Hobbs M.J., Anderson T.M., Andrews G. (2012). Psychological distress across the lifespan: Examining age-related item bias in the Kessler 6 Psychological Distress Scale. Int. Psychogeriatr..

[35] Fassaert T., Wit M.A.S.D., Tuinebreijer W.C., Wouters H., Verhoeff A.P., Beekman A.T.F., Dekker J. (2010). Psychometric properties of an interviewer-administered version of the Kessler Psychological Distress scale (K10) among Dutch, Moroccan and Turkish respondents. Int. J. Methods Psychiatr. Res..

[36] Anderson T.M., Sunderland M., Andrews G., Titov N., Dear B.F., Sachdev P.S. (2013). The 10-item Kessler psychological distress scale (K10) as a screening instrument in older individuals. Am. J. Geriatr. Psychiatry.

[37] Mcnamara B.J., Banks E., Gubhaju L., Williamson A., Joshy G., Raphael B., Eades S.J. (2014). Measuring psychological distress in older Aboriginal and Torres Strait Islanders Australians: A comparison of the K-10 and K-5. Aust. N. Z. J. Public Health.

[38] Barry L.C., Soulos P.R., Murphy T.E., Kasl S.V., Gill T.M. (2013). Association between indicators of disability burden and subsequent depression among older persons. J. Gerontol..

[39] Dixon-Ibarra A., Horner-Johnson W. (2014). Disability status as an antecedent to chronic conditions: National health interview survey, 2006–2012. Prev. Chronic Dis..

[40] Jean A., Elaine S., Daniel N., Milne E.M.G., Carol J. (2015). Anticipated survival and health behaviours in older English adults: Cross sectional and longitudinal analysis of the English Longitudinal Study of Ageing. PLoS ONE.

[41] Teesson M., Mitchell P.B., Deady M., Memedovic S., Slade T., Baillie A. (2011). Affective and anxiety disorders and their relationship with chronic physical conditions in Australia: Findings of the 2007 National Survey of Mental Health and Wellbeing. Aust. N. Z. J. Psychiatry.

[42] Husky M.M., Mazure C.M., Paliwal P., Mckee S.A. (2008). Gender differences in the comorbidity of smoking behavior and major depression. Drug Alcohol Depend..

[43] Xiang Y.-T., Wang G., Guo T., Hu C., Ungvari G.S., Kilbourne A.M., Lai Y.C., Wong Y.S., Si T.-M., Zheng Q.-W. (2013). Gender differences in demographic and clinical features and prescribing patterns of psychotropic medications in patients with major depressive disorder in China. Compr. Psychiatry.

[44] National Bureau of Statistics (2013). China Statistical Yearbook.

[45] Feng-Rong Z., Xue J. (2013). Multivariate analysis of factors affecting women’s mental health. J. Fujian Jiangxia Univ..

[46] Molarius A., Berglund K., Eriksson C., Lambe M., Nordstrom E., Eriksson H.G., Feldman I. (2007). Socioeconomic conditions, lifestyle factors, and self-rated health among men and women in Sweden. Eur. J. Public Health.

[47] Boyd C.M., Fortin M. (2010). Future of multimorbidity research: How should understanding of multimorbidity inform health system design?. Public Health Rev..

[48] Xu Q.-Q. (2019). Study on the Relationship between Mental Health and Quality of Life of Female Diabetic Patients in Shandong Province. Master’s Thesis.

[49] Xu A.-Q. (2004). Women’s physical and mental health and its influencing factors: A report from Shanghai. Collect. Women’s Stud..

[50] Wei C.-Y., Xu L.-Z., Wang J. (2019). Health poverty alleviation policies from the perspective of multidimensional poverty theory: Taking Shandong Province as an example. Shandong Soc. Sci..

